# Peripheral Neuropathy: A Phenotype-Driven Review for Diagnosis and Management

**DOI:** 10.31083/RN48384

**Published:** 2026-04-23

**Authors:** Tomoo Yuba, Ali Dabbagh, A. Sassan Sabouri

**Affiliations:** ^1^Assistant Professor, Department of Anesthesiology and Intensive Care, Osaka University Hospital, 565-0871 Osaka, Japan; ^2^MD, Professor, Anesthesiology Research Center, Department of Anesthesiology, Faculty of Medicine, Shahid Beheshti University of Medical Sciences, 4739 Tehran, Iran; ^3^Assistant Professor of Anesthesiology, Harvard Medical School, Boston, MA 02115, USA; ^4^Anesthesiologist, Department of Anesthesiology, Mass General Brigham, Boston, MA 02114, USA; ^5^Visiting Professor, Department of Anesthesiology, Shahid Beheshti Medical Science University, 4739 Tehran, Iran

**Keywords:** peripheral nervous system diseases, diabetic neuropathies, small-fiber neuropathy, ultrasonography, electromyography, nerve conduction studies, electric stimulation therapy

## Abstract

The purpose of this narrative review is to provide a clinic-ready synthesis of contemporary concepts in peripheral neuropathy, spanning epidemiology, diagnosis, and treatment, with emphasis on high-yield advances applicable to daily practice. The authors integrate pragmatic tools—including a diagnostic algorithm, suggested initial laboratory panels, and commonly used outcome measures—to support clinical decision-making. However, this review is intended as a clinic-oriented synthesis rather than a formal practice guideline. Peripheral neuropathy can be systematically categorized into seven pathophysiologic phenotypes—(1) distal “dying-back” axonopathy, (2) neuronopathy (ganglionopathy), (3) demyelinating neuropathies, (4) small-fiber neuropathy, (5) autonomic neuropathy, (6) ischemic/infiltrative/inflammatory axonopathies, and (7) focal compressive/entrapment neuropathies. An organized evaluation and management around this phenotype-first structure, combined with a structured stepwise escalation algorithm (from bedside pattern recognition to targeted laboratory testing, electrodiagnostics, selective imaging, small-fiber assessment, and immune work-up when indicated), bridges fragmented evidence into a clinic-ready decision-support framework that improves diagnostic precision, rational test utilization, and therapeutic alignment. Beyond optimizing pharmacologic care, neuromodulation may expand options in carefully selected patients. For painful diabetic peripheral neuropathy (DPN), high‑frequency (10 kHz) spinal cord stimulation (SCS) has been evaluated in randomized comparative studies against optimized medical management and has been associated with sustained pain reduction and functional improvement through 24 months in follow‑up reports, supporting consideration in medication‑refractory cases where access and patient factors permit. Ultrasound ‑guided pulsed radiofrequency (PRF)—a nondestructive, field‑based neuromodulation that limits tip temperature to <42 °C—has been studied in small randomized trials and observational cohorts for focal entrapment‑type neuropathic pain after positive diagnostic blocks; reported benefits are generally short‑ to mid‑term with heterogeneous protocols, so certainty varies by indication. For hereditary transthyretin amyloid polyneuropathy (ATTRv), disease‑modifying approaches—including nucleic acid–based therapies—are increasingly integrated into contemporary care. Overall, these developments support earlier pattern recognition, more precise phenotyping, and rational escalation while using standardized outcome measures to track response.

## 1. Introduction

Peripheral neuropathy is common worldwide, with an estimated general-population prevalence of about 1–3% that rises to roughly 7–8% in older adults [1]. In the United States, monofilament-based estimates suggest that among adults ≥40 years, overall prevalence is ~13–14%, including 28% in those with diabetes and ~12% in those without (≈18.6 million adults in 2010) [1, 2]. In Japan, contemporary registry data in type 2 diabetes show diabetic symmetric sensorimotor polyneuropathy in ~36% of patients, underscoring the substantial burden in clinical practice. Globally, diabetic peripheral neuropathy (DPN) remains one of the most frequent diabetes complications, with prevalence varying by population, duration of diabetes, and diagnostic method, often ranging from ~6% to >40% in clinical and epidemiologic studies [2]. These figures justify a pragmatic, stepwise diagnostic approach and early risk-factor modification in primary and specialty care [1, 2].

“Peripheral neuropathy” spans hundreds of etiologies and diverse phenotypes (large- vs small-fiber, axonal vs demyelinating, length-dependent vs non–length-dependent), complicating front-line recognition without a unifying, phenotype-first framework [1, 2]. Up-to-date summaries for distal sensory polyneuropathies further highlight these diagnostic nuances [3].

Peripheral neuropathy persistently impairs function and quality of life through pain, paresthesia, weakness, and autonomic symptoms, and is linked to falls, foot ulcers, work and caregiving burden, and increased healthcare utilization [1, 2]. Etiologies span metabolic (diabetes/dysglycemia), immune-mediated, genetic, toxic/drug-induced, ischemic/vasculitic and infiltrative (e.g., amyloidosis) processes, and focal compressive/entrapment disorders, while the underlying pathophysiology maps to distal “dying-back” axonopathy, neuronopathy (ganglionopathy), segmental demyelination, small-fiber and autonomic involvement, and mixed focal versus diffuse patterns [1, 2, 3]. This heterogeneity often delays diagnosis and treatment, underscoring the need for structured evaluation pathways and therapeutic choices grounded in contemporary evidence [1, 2].

Clinicians need a concise, phenotype-first synthesis that links epidemiology with a pragmatic, stepwise diagnostic pathway and an updated treatment armamentarium, including when and how to escalate from core labs and electrodiagnostic studies (EDX; nerve conduction studies [NCS] and electromyography [EMG]) to ultrasound (US) or magnetic resonance neurography (MRN), and when to consider neuromodulation or disease-modifying therapies.

**Fig. 1.**
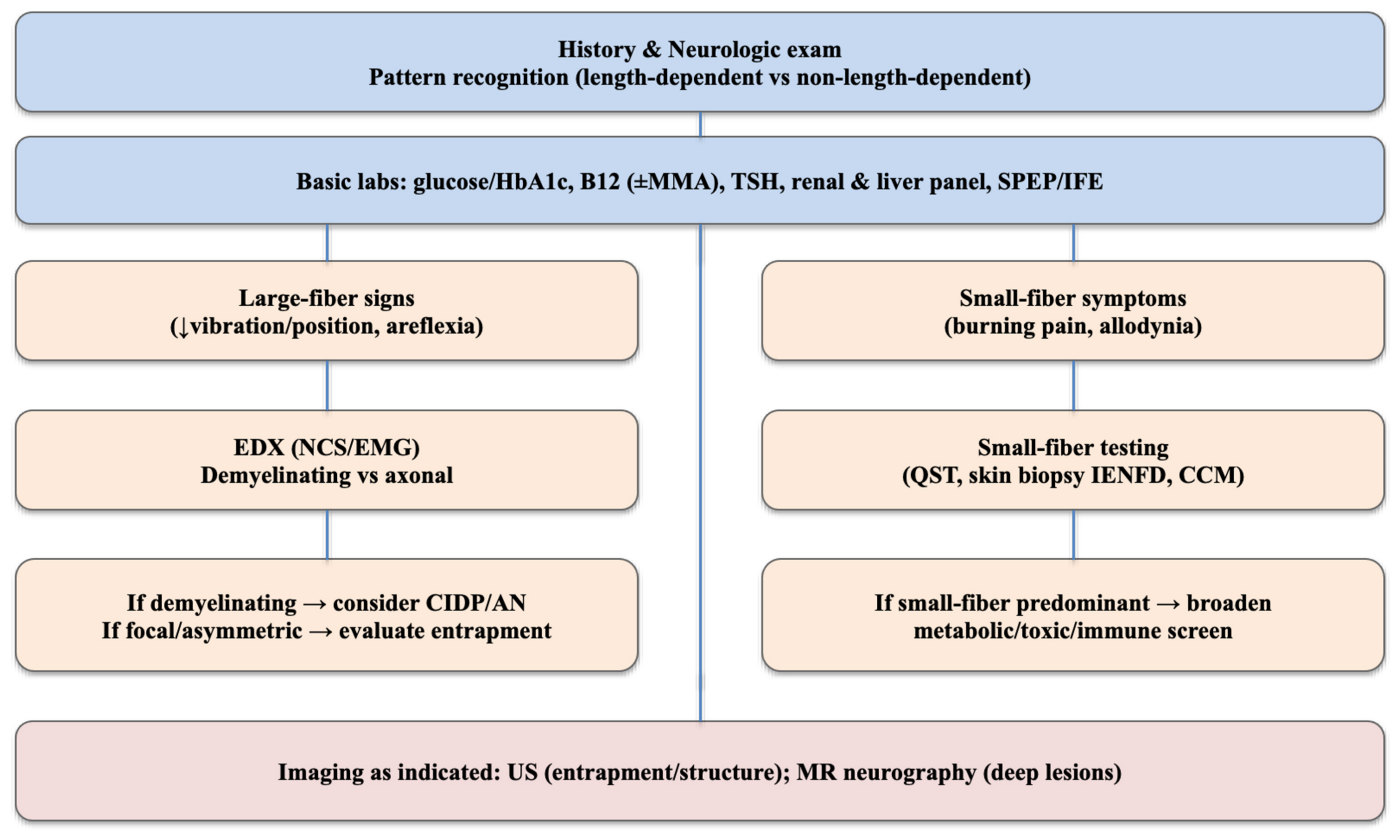
**Pragmatic diagnostic algorithm for peripheral neuropathy**. A stepwise pathway from history and focused neurologic examination to pattern recognition (length-dependent vs non–length-dependent; small- vs large-fiber), core laboratory testing (glucose/HbA1c, B12 ± MMA, TSH, renal and hepatic panels, SPEP/IFE), and electrodiagnostics (NCS/EMG) to define demyelinating vs axonal physiology. Conditional branches prompt small-fiber testing (QST, skin biopsy for IENFD, CCM) and targeted imaging (nerve US for entrapment/structural questions; MRN for deep nerves, plexus, tumor, or long-segment trauma). Decision points highlight when to suspect CIDP/AN, entrapment neuropathies, or broader metabolic/toxic/immune etiologies. Abbreviations: EDX, electrodiagnostic studies (nerve conduction studies [NCS] and electromyography [EMG]); QST, quantitative sensory testing; IENFD, intraepidermal nerve fiber density; CCM, corneal confocal microscopy; US, ultrasound; MRN, magnetic resonance neurography; AN, autoimmune nodopathies; CIDP, chronic inflammatory demyelinating polyneuropathy; MMA , methylmalonic acid.

In this review, we harmonize dispersed guidance, highlight high-yield advances (e.g., small-fiber/autonomic testing and corneal confocal microscopy), and supply a practical tool to support this knowledge gap in decision-making. We emphasize a phenotype-driven workflow and integrate clinic-facing tools (Fig. 1; Tables 1,2) as a flexible framework. We also highlight recent practice-relevant advances in imaging (high-resolution nerve US and MRN), small-fiber biomarkers, and selected device-based therapies, while explicitly grading the certainty of evidence and distinguishing established recommendations from investigational domains.

**Table 1.** **Initial laboratory panel and triggers for extended work-up**.

Tier	Items	Notes
Initial (all)	Glucose/HbA1c; B12 (± MMA); TSH; renal & liver function; SPEP/IFE	Covers common reversible causes
Conditional/extended	OGTT; autoimmune panel; HIV/HBV/HCV; folate; copper; genetic tests (as indicated)	Selected based on history/exam and basic results
When to escalate	Rapid progression; asymmetry; prominent motor deficits; systemic red flags	Consider imaging, CSF, nerve US/biopsy in select cases

Core tests used at presentation (e.g., glucose/HbA1c ± OGTT, vitamin B12 ± MMA, TSH, renal and hepatic panels, SPEP/IFE) and clinical features that trigger escalation (e.g., rapid progression, marked asymmetry, prominent weight loss or systemic signs, non—length-dependent distribution, severe autonomic involvement, or suspected immune/paraneoplastic disease). The table is intended to align the minimal primary-care panel with timely specialist escalation. Abbreviations: HbA1c, hemoglobin A1c; B12, vitamin B12; MMA, methylmalonic acid; TSH, thyroid-stimulating hormone; SPEP/IFE, serum protein electrophoresis/immunofixation electrophoresis; OGTT, oral glucose tolerance test; HIV, human immunodeficiency virus; HBV, hepatitis B virus; HCV, hepatitis C virus; CSF, cerebrospinal fluid; US, ultrasound.

**Table 2.** **Common outcome measures in neuropathy (domains and typical use)**.

Measure	Domain	Typical use
NIS-LL/mNIS+7	Impairment	Clinical trials; disease modification
R-ODS	Disability/Function	Daily function/Rasch-scaled
TNS/TNSr	Composite severity	CIPN; clinical monitoring
CNFD/CNBD/CNFL	Small-fiber	Early regeneration; 1–8 month sensitivity
IENFD (skin biopsy)	Small-fiber	Diagnosis/longitudinal change
NRS (or VAS); BPI	Pain intensity, QoL	Symptom tracking/responder analysis
QoL-DN	QoL	Diabetes-related neuropathy trials
SF-36/EQ-5D	QoL	Health utility/QALY inputs
6MWT/9HPT	Performance	Gross/hand function (selected cohorts)

This table collates validated instruments across key domains—impairment/disability (NIS-LL, mNIS+7, R-ODS), composite severity (TNS/TNSr), small-fiber structure (CNFD/CNBD/CNFL), pain/symptoms and quality of life (VAS/NRS, BPI, QoL-DN, SF-36, EQ-5D), and performance (6MWT, 9HPT)—to standardize response tracking in clinical practice and trials. Typical use cases include disease-modification readouts (NIS-LL/mNIS+7), functional follow-up (R-ODS), CIPN severity monitoring (TNS/TNSr), early regeneration signals within 1–8 months (CCM), diagnosis and longitudinal small-fiber change (IENFD), symptom and interference profiling (VAS/NRS; BPI), health-utility estimation for cost-effectiveness (SF-36; EQ-5D), and gross/hand function testing in selected cohorts (6MWT; 9HPT). Abbreviations: NIS-LL, Neuropathy Impairment Score – Lower Limbs; mNIS+7, modified Neuropathy Impairment Score +7; R-ODS, Rasch-built Overall Disability Scale; TNS, Total Neuropathy Score; TNSr, reduced Total Neuropathy Score; CNFD, Corneal Nerve Fiber Density; CNBD, Corneal Nerve Branch Density; CNFL, Corneal Nerve Fiber Length; IENFD, Intraepidermal Nerve Fiber Density; NRS, Numerical Rating Scale; VAS, Visual Analogue Scale; BPI, Brief Pain Inventory; QoL-DN, Quality of Life – Diabetic Neuropathy; SF-36, Short Form 36; EQ-5D, EuroQol 5-Dimension; 6MWT, 6-Minute Walk Test; 9HPT, 9-Hole Peg Test; QoL, Quality of Life; QALY, Quality-Adjusted Life Year; CIPN, Chemotherapy-Induced Peripheral Neuropathy.

Although this narrative review synthesizes clinically actionable concepts across major neuropathy phenotypes, it does not constitute a formal clinical practice guideline.

Objectives of this narrative review are : (1) to present a phenotype-first approach that helps clinicians localize and classify neuropathy at the bedside; (2) to summarize a practical, stepwise diagnostic pathway—including when to escalate from core laboratory testing and EDX to US/MRN and small-fiber evaluation; (3) to provide an evidence-anchored overview of contemporary management options across major etiologies, with clear boundaries between guideline-based care and emerging interventions.

Intended audience:

While the framework is designed to be accessible and practical for general medical professionals, particular emphasis is placed on neurology and pain medicine practitioners, for whom detailed diagnostic escalation and interventional considerations may be especially relevant.

What is New in this review: This review is the integration of a phenotype-first approach with a structured, stepwise diagnostic escalation algorithm that translates fragmented evidence into a practical, clinic-ready workflow. Unlike traditional etiology-based summaries, this review systematically links bedside pattern recognition to targeted laboratory testing, electrodiagnostics, selective use of high-resolution US and MRN, small-fiber assessment (including corneal confocal microscopy), and nodal/paranodal antibody evaluation when indicated. It also aligns contemporary therapeutic options—guideline-based pharmacotherapy, image-guided interventions, and neuromodulation—within the same framework, explicitly distinguishing established evidence from emerging domains.

## 2. Method

This is a narrative (non-systematic) review formed by targeted searches of PubMed/MEDLINE (https://pubmed.ncbi.nlm.nih.gov/), supplemented by citation tracking in Google Scholar (https://scholar.google.com/). We focused primarily on 2010–2025 to capture contemporary diagnostic technologies (US/MRN), biomarkers, and device-based therapies, while also incorporating landmark pre‑2010 evidence when it emphasizes current standards (typically via major guidelines/consensus statements or high-quality syntheses). Article selection was purpose-driven: we prioritized (i) current professional-society guidelines/consensus statements for screening and management statements, (ii) systematic reviews/meta-analyses and randomized trials for practice-facing interventions, and (iii) representative observational studies and authoritative reviews when higher-level evidence was limited. We excluded single case reports and purely preclinical studies from practice recommendations (they are discussed only as mechanistic or investigational context). Our targeted searches returned several hundred records. After title/abstract screening and prioritization, we reviewed ~100 full texts; 60 key sources were ultimately cited. When evidence was conflicting, we favored higher-quality designs, consistency across studies, and alignment with guideline positions, and we explicitly qualify certainty where data remain heterogeneous.

## 3. Classification & Pathophysiology

Peripheral neuropathies can be organized into seven pathophysiologic categories that map directly to clinical presentation and testing strategy: (1) distal “dying-back” axonopathy, (2) neuronopathy (ganglionopathy), (3) demyelinating neuropathies, (4) small-fiber neuropathy, (5) autonomic neuropathy, (6) ischemic/infiltrative/inflammatory axonopathies, and (7) focal compressive/entrapment neuropathies.

This classification is used to guide a phenotype-first diagnostic strategy and escalation of testing.

### 3.1 Axonal Degeneration (Distal “Dying‑back” Axonopathy)

Distal symmetric polyneuropathy (DSP) is most often explained by length-dependent metabolic or toxic axonal injury, with typical Wallerian and distal axonopathy patterns. Clinically, it presents as symmetric, length-dependent sensory more than motor deficits, usually chronic in course. Electrodiagnostic testing reveals reduced compound muscle action potential (CMAP) and sensory nerve action potential (SNAP) amplitudes with relatively preserved conduction velocities and latencies. Common causes include diabetes and other dysglycemic states, alcohol use, toxins and medications, nutritional deficiencies, and renal or hepatic disease, though many cases remain idiopathic and axonal in nature [4].

### 3.2 Neuronopathy (Ganglionopathy) — Primary Neuronal Cell‑Body Loss

Sensory neuronopathies arise from injury to the dorsal root ganglion, or less commonly the anterior horn cell, and differ from typical peripheral neuropathies by their non–length-dependent and often asymmetric distribution. Clinically, they present with sensory loss and marked sensory ataxia while motor strength is relatively preserved early in the disease course. Electrodiagnostic studies show diffusely reduced or absent sensory responses with relatively preserved motor conduction. These disorders are most often associated with autoimmune conditions such as Sjögren syndrome, paraneoplastic syndromes, and certain toxins including platinum-based chemotherapeutic agents [5].

### 3.3 Demyelinating Neuropathies (Segmental Demyelination)

Demyelinating neuropathies are caused by injury to the myelin sheath, resulting in conduction slowing and block, and may occur as acquired immune-mediated or hereditary disorders. They typically present with proximal and distal weakness and areflexia, with a tempo that ranges from acute onset, as in Guillain–Barré syndrome, to chronic progression, as seen in chronic inflammatory demyelinating polyradiculoneuropathy (CIDP). Electrodiagnostic studies reveal slowed conduction velocities, prolonged distal latencies, temporal dispersion, conduction block, and abnormal or absent F-waves. Etiologies include the CIDP spectrum and other immune-mediated neuropathies, paraproteinemic forms, and hereditary demyelinating conditions such as Charcot–Marie–Tooth type 1. This category includes both acquired demyelinating neuropathies and hereditary demyelinating neuropathies (e.g., Charcot–Marie–Tooth disease [CMT], hereditary neuropathy with liability to pressure palsies [HNPP]) [6].

### 3.4 Small-Fiber Neuropathy (Small Myelinated A-δ and Unmyelinated C Fibers)

Small-fiber neuropathy arises from selective injury to nociceptive and autonomic fibers, producing a clinical pattern dominated by burning pain, allodynia, thermal dysesthesias, and frequent autonomic symptoms, while examination and standard nerve conduction studies may remain normal. Diagnosis relies on specialized testing, most notably skin biopsy to assess intraepidermal nerve fiber density and autonomic evaluations such as the quantitative sudomotor axon reflex test (QSART) or heart rate variability. Common etiologies include dysglycemia and diabetes, amyloidosis, sarcoidosis, Sjögren syndrome, various toxins, and idiopathic causes [7].

### 3.5 Autonomic Neuropathy

Autonomic neuropathy involves injury to sympathetic and parasympathetic fibers, often overlapping with small-fiber disease, and is characterized clinically by orthostatic hypotension, gastrointestinal dysmotility, genitourinary dysfunction, and sudomotor abnormalities, with diabetes frequently amplifying morbidity. Diagnosis relies on standardized autonomic function testing performed in conjunction with somatic nerve assessments. The most recognized context is diabetic cardiovascular autonomic neuropathy, along with other diabetic autonomic syndromes [8].

### 3.6 Ischemic/Infiltrative/Inflammatory Axonopathies (Mixed Mechanisms)

Vasculitic neuropathy results from ischemic infarction of the vasa nervorum and typically presents as a subacute, painful, asymmetric mononeuropathy multiplex, where urgent recognition and treatment can alter outcomes. Amyloid and other infiltrative neuropathies arise from extracellular deposition, often producing a length-dependent phenotype with prominent small-fiber and autonomic features. Toxic–inflammatory neuropathies, such as those induced by chemotherapy, usually manifest as axonal, length-dependent sensorimotor involvement [9].

### 3.7 Focal Compressive/Entrapment Neuropathies (Localized Demyelination ± Secondary Axonal Loss)

Entrapment neuropathies arise from mechanical compression and ischemia at common entrapment sites, producing early demyelination with potential axonal loss if injury is severe or prolonged. Clinically, they present with focal territorial deficits, such as median neuropathy at the wrist, and may coexist with systemic polyneuropathies, particularly in the context of diabetes [10].

Overlap among neuropathic disorders is common, as many conditions evolve to produce mixed axonal and demyelinating features over time, while autonomic and small-fiber involvement frequently coexist and contribute to the overall clinical presentation.

## 4. Diagnostic Algorithm

Diagnostic algorithm outlines a practical decision pathway—progressing from initial assessment to ancillary testing, imaging, small-fiber evaluation, and immune work-up—to clarify what should be done, when, and why. The process is guided by five core pillars: (1) early recognition of red flags; (2) structured pattern recognition (length-dependent vs non–length-dependent, symmetric vs asymmetric, small- vs large-fiber); (3) appropriate selection and sequencing of ancillary tests; (4) judicious use of imaging and small-fiber diagnostic tools; and (5) vigilance for immune-mediated etiologies, particularly nodal and paranodal antibody–associated disease (Fig. 1).

### 4.1 History & Examination

Symmetric, predominantly motor weakness that evolves over ≤4 weeks with reduced/absent reflexes may prompt consideration of Guillain–Barré syndrome (GBS) and expedited decisions regarding intravenous immunoglobulin (IVIg) or plasma exchange (PE) [11]. Dysphagia, respiratory compromise, or rapidly evolving autonomic instability warrants intensive care unit (ICU)-level monitoring [11]. By contrast, a painful, asymmetric mononeuritis multiplex–like picture with systemic inflammatory features should raise concern for vasculitis or paraneoplastic disease and trigger early specialty referral for electrodiagnostics, serology, and—when appropriate—biopsy.

A symmetric, length-dependent, sensory-predominant presentation points first to metabolic/toxic causes such as DPN or chemotherapy-induced peripheral neuropathy (CIPN) [12, 13]. Non–length-dependent or multifocal patterns raise the likelihood of immune-mediated neuropathies (e.g., CIDP or autoimmune nodopathies), vasculitis, or paraneoplastic etiologies [14]. A small-fiber–predominant syndrome—burning pain with thermal/pinprick loss and relatively preserved vibration—suggests early DPN, CIPN, or amyloid and should prompt small-fiber testing [12, 13]. Chronic (≥8 weeks) symmetric sensorimotor deficits with reduced reflexes keep CIDP or nodopathy high on the list [14].

Bedside screening remains valuable: thermal/pinprick for small- fibers; 128-Hz tuning fork and joint-position sense for large fibers; and 10-g monofilament for protective sensation. For DPN, at least annual neuropathy assessment is advised (type 2: from diagnosis; type 1: from 5 years after onset) [12].

### 4.2 Labs & Electrodiagnostics

Clinicians are encouraged to ensure that laboratory testing and electrodiagnostic studies are pattern-driven rather than ordered as a “shotgun” panel. When DPN is likely, prioritize optimization of metabolic control; when immune disease is suspected, EDX and targeted inflammatory/immune labs. In CIPN, document the drug history, dose, and cumulative exposure [12, 13].

EDX, including NCS and EMG, are pivotal to define (1) demyelinating vs axonal physiology; (2) length-dependent vs non–length-dependent involvement; (3) the presence of conduction block (CB) and temporal dispersion; and (4) anatomic localization. In CIDP, typical demyelinating features include prolonged distal latencies, slowed conduction velocities, CB/temporal dispersion, and prolonged F-waves, findings that directly inform treatment decisions. In autoimmune nodopathies, watch for prominent CB with little or no temporal dispersion, consistent with reversible conduction failure at the node/paranode [14]. CIPN most often shows a sensory-predominant axonal pattern [13].

For carpal tunnel syndrome (CTS) as a focal confirmation scenario, a paired-accuracy meta-analysis indicates similar diagnostic performance for nerve-muscle US and EDX (sensitivity 86% vs 92%, specificity 79% vs 82%); shared decision-making can weigh patient preference, cost, and availability [15].

### 4.3 Imaging

US and MRN serve complementary roles; selecting the modality based on its specific strengths enhances diagnostic yield. US is favored for superficial nerves, small branches, dynamic compression, and metal-adjacent regions (e.g., near postoperative hardware or devices). It visualizes extrinsic compression, gliding impairment, cysts/foreign bodies in real time, and is rapid, bedside-capable, and cost-effective [16, 17]. MRN is central for deep nerves, brachial/lumbosacral plexus, tumors, and extensive trauma. When available, use higher field strength and optimized 3D isotropic protocols with robust fat suppression and suppression of intravascular signal to improve conspicuity of nerve fascicles and perineural pathology [18, 19]. In the presence of metal, artifact mitigation can include using lower field strength and metal artifact–reduction techniques with appropriate bandwidth adjustments [16, 18].

Operationally, in entrapment neuropathies, start with US to detect swelling/flattening, hyperemia, and dynamic signs; add MRN when needed to map the full extent and denervation changes [16, 19]. In trauma/postoperative settings, US is advantageous near sutures and hardware, whereas MRN best captures the overall extent of plexus or long-segment injuries [16, 18]. For tumor/infiltration, MRN depicts features of peripheral nerve sheath tumors (PNST) (e.g., fascicular sign/target sign) and can suggest malignancy (e.g., lower apparent diffusion coefficient (ADC)); consider fluorodeoxyglucose positron emission tomography (FDG-PET) when appropriate [16].

### 4.4 Small-Fiber Assessment

When small-fiber–predominant involvement is suspected (burning pain, thermal hypoalgesia, prominent paresthesia with largely normal EDX), combine intraepidermal nerve fiber density (IENFD) from skin biopsy (invasive but standardized histologic metric), quantitative sensory testing (QST) (functional but subject-dependent), and corneal confocal microscopy (CCM) (noninvasive quantitative imaging). Of these, CCM has shown early sensitivity to regeneration, detecting significant increases in corneal nerve fiber density (CNFD), corneal nerve fiber branch density (CNBD), and corneal nerve fiber length (CNFL) as soon as 1–8 months after pharmacologic or surgical interventions, with effects sustained longer term—supporting its role as a biomarker of early small-fiber recovery and a bridge between trials and practice [20].

Mechanistic evidence suggests that hyperglycemia/metabolic stress can alter exosome release and cargo (e.g., miRNA/protein/lipid profiles), thereby amplifying neuroinflammation, Schwann-cell dysfunction, impaired axonal regeneration, and microvascular injury—processes central to diabetic neuropathy pathogenesis. In contrast, clinical evidence remains preliminary: circulating exosomal signatures (including candidate miRNAs proposed in early studies) may serve as minimally invasive biomarkers for early detection, phenotyping (painful vs painless), and longitudinal monitoring, but require standardized isolation/assays and prospective validation across cohorts. Therapeutically, most data are preclinical, where Schwann cell–derived or mesenchymal stromal cell–derived exosomes have been reported to support neuroprotection and repair (mitochondrial support, inflammation modulation, axonal regeneration), and engineered exosomes are being explored as targeted delivery vehicles for therapeutic nucleic acids or drugs; however, translation is limited by manufacturing scale-up, cargo consistency, dosing/route optimization, and safety/efficacy confirmation in human trials [21].

Despite its promise, CCM (and other specialized small-fiber assessments) has limited availability in routine practice, requires dedicated equipment and trained expertise, and demonstrates variability in normative values and diagnostic thresholds across devices, acquisition protocols, and reference populations [22].

### 4.5 Autoimmune Nodopathies (Antibody Testing)

Testing for nodal/paranodal antibodies should be guided by specific clinical triggers. Evaluation is warranted in patients with CIDP-like disease who demonstrate a poor or early-relapsing response to IVIg; prominent ataxia, coarse tremor, or marked sensory ataxia; cranial, ocular, or respiratory muscle involvement; electrodiagnostic studies showing prominent conduction block with minimal temporal dispersion suggestive of nodal dysfunction; or magnetic resonance imaging (MRI)/MRN evidence of nerve root or plexus hypertrophy [14].

Recommended testing includes antibodies to neurofascin-155 (NF155), neurofascin-186 (NF186), neurofascin-140 (NF140), contactin-1 (CNTN1), and contactin-associated protein 1 (CASPR1) (including pan-neurofascin). Cell-based assays (CBA) are preferred, as ELISA alone may yield false-positive or false-negative results; accordingly, CBA-focused diagnostic strategies are advised [14]. IgG subclass may have therapeutic implications. Immunoglobulin G (IgG) -predominant responses (e.g., against NF155, CNTN1, or CASPR1) are often less responsive to IVIg and may respond better to B-cell–targeted therapies such as rituximab. In contrast, acute IgG3-predominant cases may initially respond to IVIg but can later switch to an IgG4 profile and become treatment-refractory [14].

### 4.6 Summary

Fig. 1 summarizes a pragmatic diagnostic algorithm for peripheral neuropathy, and Table 1 outlines the core initial and conditional tests used in this review.

A practical approach is to: (1) triage red flags first (GBS, autonomic/respiratory compromise) [11]; (2) apply pattern recognition (length-dependent vs non–length-dependent; symmetric vs asymmetric; small- vs large-fiber); (3) use core labs and EDX to establish axonal vs demyelinating vs nodal physiology [12, 13, 14]; (4) select imaging appropriately—US for superficial/dynamic/metal-adjacent scenarios, MRN for deep nerves, plexus, tumors, and extensive trauma—with optimized protocols for the clinical question and metal artifact–reduction strategies when needed [16, 18, 19]; (5) when small-fiber involvement is likely, incorporate CCM/IENFD/QST, noting CCM’s sensitivity to early regeneration [20]; and (6) if IVIg-refractory CIDP-like disease or nodal signatures are present, test by CBA for NF155/CNTN1/CASPR1 (± pan-neurofascin (pan-NF)) and align treatment accordingly [14]. For focal entrapment (e.g., CTS), US and EDX offer comparable accuracy, so shared decision-making based on preference, cost, and access is appropriate [15].

Clinicians may consider escalating the diagnostic workup to EDX (NCS/EMG) when large-fiber involvement is suspected (objective sensory loss, weakness, areflexia), when the pattern is asymmetric, non–length-dependent, or rapidly progressive, when an immune-mediated neuropathy is suspected, or when the diagnosis remains unclear after core laboratory testing (and before initiating immunotherapy). Proceed to US when symptoms suggest focal entrapment/structural pathology, when dynamic compression is suspected, when evaluation is needed near metal/hardware, or when pre-procedural planning is required (e.g., for targeted injection or surgical planning). Proceed to MRN when deep nerves or plexus are suspected to be involved, when tumor/trauma or extensive postoperative injury is suspected, when symptoms remain unexplained despite EDX/US, or when whole-course mapping is required for management decisions. Proceed to genetic testing when onset is in childhood/early adulthood, when there is a family history or characteristic skeletal deformity (e.g., pes cavus), or when EDX suggests a hereditary pattern (e.g., diffuse uniform demyelination without clear conduction block or recurrent pressure palsies). Proceed to biopsy when results are likely to change management—particularly in suspected vasculitic neuropathy (painful, asymmetric mononeuritis multiplex with systemic inflammatory features), suspected amyloidosis/infiltrative neuropathy, or suspected small-fiber neuropathy with normal EDX (skin biopsy for IENFD as appropriate) [9].

In children, the algorithm requires several pediatric-specific safeguards. Red flags include delayed motor milestones, gait clumsiness, pes cavus, scapular winging, and unexplained pain crises or autonomic instability. Time course still guides the differential—acute/subacute patterns often reflect infectious or post-infectious causes (including pediatric GBS variants), whereas chronic courses suggest toxic/metabolic or hereditary etiologies. Initial testing mirrors adults but prioritizes potentially reversible causes (e.g., B12 deficiency, thyroid disease, inflammatory screens) and a careful drug/toxin review; when phenotype or family history points to a genetic disorder, early targeted genetics is appropriate. Electrodiagnostic testing should use age-adjusted norms, and imaging (US or MRN) helps exclude structural entrapment and can inform biopsy decisions [11, 23]. Management emphasizes physiotherapy and orthotics, child-appropriate analgesic dosing, and family genetic counseling; immunotherapy follows established pediatric GBS/CIDP frameworks when indicated. Overall, apply the same stepwise logic, but lower the threshold for genetics, adjust EDX interpretation for age, and focus on reversible causes early.

## 5. Treatment: Pharmacologic to Neuromodulation

To clarify the clinical intent, we group treatments into (i) symptomatic management, (ii) etiology-directed (disease-modifying) therapy when available, and (iii) investigational or emerging approaches; statements are phrased cautiously when evidence is limited.

This section also outlines a three-step clinical decision pathway for painful peripheral neuropathy—pharmacologic therapy, image-guided interventions, and stepwise introduction of neuromodulation for refractory pain—emphasizing optimization of first-line medications and selective, minimally invasive procedures when appropriate.

Common outcome metrics used to track response in both trials and practice are summarized in Table 2.

When tracking response across domains, a practical option is to use outcome measures that span impairment, disability, quality of life, and pain, and—where available—small-fiber structure. Examples include Neuropathy Impairment Score–Lower Limbs (NIS-LL), modified NIS+7 (mNIS+7), Rasch-built Overall Disability Scale (R-ODS), Norfolk Quality of Life–Diabetic Neuropathy (QoL-DN), Total Neuropathy Score (TNS/TNSr), and pain measures such as Numeric Rating Scale (NRS) or Brief Pain Inventory (BPI), with IENFD or CCM used in specialized settings to capture small-fiber structure [24, 25, 26].

### 5.1 Symptomatic Management: Pharmacologic Therapy

For painful diabetic peripheral neuropathy (PDN) and other neuropathic pain syndromes, first-line pharmacologic options include serotonin–norepinephrine reuptake inhibitors (SNRIs), tricyclic antidepressants (TCAs), gabapentinoids, and sodium channel blockers, all supported by moderate-quality evidence [27, 28]. Across high-quality guidelines and meta-analyses, SNRIs (e.g., duloxetine), gabapentinoids (gabapentin/pregabalin), and sodium-channel blockers (e.g., mexiletine, carbamazepine; lamotrigine for selected phenotypes) achieve broadly similar small-to-moderate average pain reductions in painful neuropathy; in practice, between-class differences are driven more by adverse-event profiles and comorbidities than by efficacy per se. Duloxetine often suits patients with co-existing depression/anxiety or fatigue; gabapentinoids warrant caution for sedation, dizziness/falls, weight gain, and the need for renal dosing; Na⁺ blockers require electrocardiogram (ECG)/QTc vigilance and drug–drug interaction review.

A pragmatic rule is to begin with one first-line class, reassess at 8–12 weeks using predefined pain and function thresholds, and—if there is partial benefit with good tolerance—either switch classes or consider low-dose combination therapy; prioritize safety and tolerability over theoretical additive efficacy. Their average effect sizes are comparable, and opioids should generally be avoided except as short-term rescue in selected cases [27]. The therapeutic goal should focus on pain reduction and functional improvement rather than complete elimination. Response is typically assessed after 8–12 weeks; partial responders may benefit from class switching or combination therapy [29].

Dosing and titration should be individualized according to sedation, fall risk, weight gain, anticholinergic burden, renal and hepatic function, and potential interactions (e.g., selective serotonin reuptake inhibitors [SSRI]/SNRI coadministration or tricyclic antidepressant (TCA) cardiotoxicity) [28]. A conservative initial dose and slow titration are recommended in elderly or renally impaired patients. When combining agents, safety and tolerance should guide the regimen rather than expecting additive efficacy [29]. As adjunctive options, the 8% capsaicin patch provides modest yet clinically meaningful analgesia with minimal systemic exposure [30].

Transient local burning is common but self-limited. Topical agents, including lidocaine 5% patch, can be used as adjuncts, particularly for patients intolerant of systemic drugs or with focal neuropathic pain [27].

### 5.2 Symptomatic Management: Interventional Pain Procedures

When pharmacotherapy provides insufficient relief, US-guided procedures may be appropriate. These require precise anatomical targeting and risk assessment, including management of anticoagulation, infection, and glycemic control [19, 31].

The use of corticosteroids should be determined on a case-by-case basis, and specific indications are outlined below. Corticosteroids are indicated in selected conditions such as Morton’s neuroma, where injection of local anesthetic combined with a small dose of non-particulate corticosteroid can provide short-term improvement in pain and function [32, 33]. For greater occipital nerve (GON) blocks, evidence is mixed in migraine (no added benefit of steroid in an randomized controlled trial (RCT)) but supportive in cluster headache [34, 35, 36].

Routine use of corticosteroids should be avoided for sympathetic blocks and simple trigger-point injections due to limited demonstrated benefit and the potential for adverse effects. Decisions should be individualized based on patient comorbidities (e.g., diabetes, infection risk) and aligned with local practice protocols [37].

For cases with suspected perineural adhesion or compression, hydrodissection using saline or dilute local anesthetic ± low-dose steroid may improve nerve mobility and pain in selected patients. Evidence is mainly derived from small observational studies and case series; therefore, short-term improvements have been reported, and hydrodissection can be considered between conservative therapy and surgical decompression on a case-by-case basis [19, 38].

### 5.3 Symptomatic Management: Neuromodulation

For patients with moderate-to-severe neuropathic pain refractory to optimized conservative therapy, neuromodulation provides a key therapeutic option. Proper patient selection—based on pain distribution, disease mechanism, psychological readiness, and comorbidities—is critical to ensure response. This section covers spinal cord stimulation (SCS) and pulsed radiofrequency (PRF), which modulate pain transmission without nerve destruction [39, 40].

#### 5.3.1 10 kHz SCS

High-frequency 10 kHz SCS is an implantable neuromodulation therapy in which one or two percutaneous epidural leads are positioned over the dorsal columns—typically mid-thoracic (around T8–T12 for painful diabetic neuropathy)—and connected subcutaneously to a pulse generator placed in the flank or upper buttock. The system delivers continuous, paresthesia-independent stimulation at 10,000 Hz with short pulse widths and low amplitudes; modern devices are programmable, often MRI-conditional, and available in rechargeable or primary-cell formats.

In medication-refractory PDN, adding 10 kHz SCS to optimized medical management produced markedly higher responder rates and quality-of-life gains than medical management alone at 6 months in a randomized trial [41], with durable benefit through 24 months, including large average pain reductions and clinically meaningful improvements in neurological examination domains [39]. These effects, together with the superiority of high over low-frequency SCS in other neuropathic pain conditions, support its positioning when first-line pharmacotherapy fails or is not tolerated [42].

Before permanent implantation, diabetes control and foot-risk management should be optimized, and a short externalized trial should confirm individual efficacy using predefined criteria (e.g., ≥50% reduction in average pain and/or functional gains in gait, sleep, or daily activity) [39, 41].

Complications include lead migration, infection, wound problems, hardware failure, and—rarely—neuraxial adverse events; infection risk around 2–6% has been reported and can be mitigated with timed perioperative antibiotics, meticulous sterile technique, occlusive dressings, early wound surveillance, careful lead anchoring, and early activity precautions [43, 44].

#### 5.3.2 PRF

PRF delivers short bursts of high-voltage current while maintaining tissue temperature below 42 °C, producing non-destructive neuromodulation that influences pain signaling and neuroinflammation [45]. Mechanistically, PRF disrupts abnormal firing, modifies cytokine expression, and attenuates central sensitization, providing selective modulation of nociceptive pathways [46, 47].

PRF is indicated for localized neuropathic pain with consistent provocation/relief pattern, positive diagnostic block, and safe access confirmed by US or fluoroscopy. It can serve as a bridge between pharmacologic therapy and SCS.

The electrode tip is positioned near the target nerve, typically with two 120-second cycles. US guidance allows precise placement while minimizing vascular contact and thermal injury. However, the parameter sets reported in this section (e.g., voltage, pulse width, frequency, cycle duration, and tip-temperature limits) are representative examples rather than a universal standard. Optimal settings vary with the anatomical target, patient factors, and device specifications (generator and electrode). At present, no internationally agreed standardized PRF protocol exists; parameters should be individualized and optimized within each institution’s procedural policies and ethical oversight.

Across small trials and observational studies, PRF has shown a generally favorable safety profile and may provide short- to medium-term pain relief for focal neuropathic pain syndromes such as carpal tunnel, occipital neuralgia, and Morton’s neuroma [48, 49, 50].

However, outcome heterogeneity persists across studies, emphasizing the need for standardized parameters and phenotype-based patient selection (e.g., small- vs. large-fiber dominance, inflammatory markers) in future controlled trials. PRF is particularly suited for superficial, well-visualized targets and can be performed as a two-step process: diagnostic block followed by PRF if temporary relief is achieved [51].

### 5.4 Investigational/Limited-Evidence Adjuncts: Cannabinoids, Ketamine, Mexiletine

Cannabinoids: evidence for chronic neuropathic pain shows small-to-moderate short-term benefit with frequent dose-limiting adverse effects (sedation, cognitive changes), variable legal access, and uncertain long-term safety; reserve for refractory cases after shared decision-making [52].

Ketamine: intravenous (IV) ketamine can provide short-term analgesia and wind-down central sensitization in highly refractory neuropathic pain; limit to monitored settings with defined protocols and psychological screening; durability and optimal maintenance remain uncertain [53].

Mexiletine: oral Na⁺ channel blocker useful for select sodium-channel–mediated pain and refractory PDN; monitor for gastrointestinal (GI) intolerance and QT-related risk; consider ECG at baseline and dose escalation. These options should not displace first-line agents and should be integrated within multidisciplinary care, with explicit risk–benefit documentation [54].

(Disease-modifying therapy) Whenever an etiology-directed option exists, addressing the underlying cause should be prioritized alongside symptom control (e.g., immune, metabolic, toxic, compressive, or systemic causes), ideally coordinated with neurology and relevant specialty services.

## 6. Perioperative & Anesthesia-Related Peripheral Neuropathy

Perioperative peripheral nerve injury (PPNI) is an umbrella term encompassing both anesthesia-related mechanisms (peripheral nerve blocks, patient positioning, injection pressure, local anesthetic neurotoxicity) and surgery-related mechanisms (direct trauma, traction, entrapment, thermal injury, and ischemia). Reported incidence across all surgeries ranges from ~0.03% to 0.5%, with most cases being transient sensory deficits; persistent symptoms after peripheral nerve block are uncommon, approximately 0.02–0.1% [55, 56]. Importantly, PPNI fits within the core diagnostic framework in this review as an acute focal neuropathy in the iatrogenic/traumatic branch (Fig. 1; Section 3): localize the deficit, triage red flags, then use targeted imaging (US/MRN) and electrodiagnostics to define mechanism and guide management. Presentations span from mild paresthesia to permanent motor loss, and multifactorial causation complicates both diagnosis and prevention.

### 6.1 Mechanisms and Pathophysiology

PPNI arises from combined mechanical compression or stretch, ischemia, injection pressure–related fascicular injury, neurotoxicity, and position-related external compression. In anesthesia, intraneural (intrafascicular) injection, excessive injection pressure, prolonged fixed positioning, and tourniquet-induced blood flow interruption are key drivers [57, 58]. Surgical contributors include traction, compression, thermal damage, and ischemia in procedures such as orthopedic (shoulder/elbow), cardiac, urologic/gynecologic, and head & neck surgery [55].

### 6.2 Common Perioperative Patterns

Common perioperative patterns include position-related neuropathies (e.g., lithotomy-associated femoral/obturator compression; prone positioning with axillary/radial compression; brachial plexus stretch with shoulder abduction), tourniquet-related ischemic injury with longer inflation times, block-related injury associated with intrafascicular injection/elevated injection pressure and higher local anesthetic exposure, and direct surgical trauma or traction (e.g., shoulder/elbow fixation or trocar-related injury to the lateral femoral cutaneous nerve) [55, 59, 60, 61] .

Regional anesthesia–associated neuropathy: After peripheral nerve block, most postoperative neurologic symptoms are transient paresthesias; persistent deficits are uncommon (0.02–0.1%). Typical presentations are sensory-predominant and map to the block territory, though motor involvement can occur with intrafascicular injection, high injection pressure, or compressive hematoma [57]. Risk factors include preexisting neuropathy (e.g., diabetes), prolonged/labored needle manipulation, large volumes or high concentrations of local anesthetic, and prolonged fixed positioning [62]. Multifactorial causation is frequent and may involve both anesthesia- and surgery-related mechanisms. During block placement, combine real-time US with low-current nerve stimulation when feasible, avoid injection against high opening pressure or patient-reported severe pain/paresthesia, and stop immediately if fascicular expansion or intraneural spread is suspected. Incorporating injection-pressure monitoring and using fractional, aspiration-interrupted injections reduce the likelihood of intrafascicular delivery. For patients with preexisting neuropathy (e.g., diabetes), adopt the lowest effective dose/volume and document baseline deficits to mitigate “double-crush” confounding.

### 6.3 PPNI Prevention Strategies

Prevention can be summarized as (1) optimize technique during regional anesthesia (US-guided needle visualization, avoid injection against high opening pressure or severe paresthesia; consider pressure monitoring as an adjunct), (2) minimize exposure (use the lowest effective dose/volume and avoid incompatible admixtures), and (3) reduce mechanical/ischemic stress (neutral positioning with periodic checks, individualized tourniquet pressure and shortest feasible duration). In selected high-risk procedures, intraoperative neuromonitoring (motor evoked potentials [MEP] and somatosensory evoked potentials [SEP]) may facilitate early detection [16, 55, 58, 59, 60, 61].

### 6.4 Postoperative Evaluation and Management

When new deficits arise, consider both anesthesia- and surgery-related etiologies. Early US or MRN helps define anatomical localization, while electrodiagnostics differentiate axonal vs. demyelinating patterns [16, 18]. In the acute phase, distinguish reversible edema from irreversible axonal injury; prompt decompression of external sources and early rehabilitation can influence outcomes. If positioning, traction, or block-related mechanisms are implicated, implement recurrence-prevention bundles (positioning protocols, US proficiency training, injection pressure alarms) at the institutional level. For continuous perineural catheters, monitor for unexpectedly prolonged or dense motor block; if present, pause infusion and reassess before resuming at lower concentration. In anticoagulated patients or those with difficult needle passes, maintain a low index of suspicion for bleeding-related compression. Signs of local infection (fever, erythema, discharge) should prompt catheter removal and appropriate cultures and antibiotics.

### 6.5 Informed Consent

Although rare, PPNI can be life-altering. Preoperative counseling should state that: (1) injuries are uncommon but not fully preventable; (2) many cases improve over weeks to months; and (3) severe cases may require prolonged rehabilitation or reconstruction. Written consent and documentation are recommended [55, 57].

## 7. Future Directions

In this section, we distinguish established evidence from emerging concepts; for emerging technologies (e.g., artificial intelligence (AI)-enabled decision support, closed-loop systems, and biomarker-anchored response criteria), wording is intentionally cautious given limited prospective validation. Established evidence: High-frequency (10 kHz) SCS provides durable pain relief and functional gains in medication-refractory painful diabetic neuropathy (PDN), with sustained benefits through 24 months [39, 41]. Emerging concepts: Neuromodulation may increasingly be considered earlier in selected peripheral neuropathies as a mechanism-linked strategy, alongside etiologic treatment where applicable.

The next decade is expected to emphasize three converging themes: precision selection, objective response tracking, and smarter devices. On the selection side, phenotyping will move beyond “painful vs painless” toward profiles that integrate small-fiber status (e.g., corneal confocal microscopy metrics such as CNFD/CNBD/CNFL) and axonal injury signals (e.g., blood neurofilament light chain [NfL]), which may enable responder enrichment and earlier referral when pharmacotherapy stalls [63, 64, 65]. For response tracking, trials and clinics will increasingly pair patient-reported outcomes with structural or physiological readouts (CCM change over 1–8 months, quantitative sensory testing, gait and actigraphy, and—where available—neurophysiologic markers), shortening the cycle between a therapeutic adjustment and demonstrable nerve recovery [63].

Device platforms are also evolving. Paresthesia-independent SCS will continue to compete with pattern-based waveforms, while PRF expands options for focal, territory-bound pain and painful entrapments that are suboptimally served by axial SCS. In parallel, US-guided percutaneous peripheral nerve stimulation (PNS) and targeted interfascial techniques may offer minimally invasive bridges between conservative care and surgery, especially when combined with standardized injection-pressure monitoring and dose minimization to protect vulnerable nerves (continuing the safety principles emphasized in perioperative practice). For inflammatory or infiltrative axonopathies, neuromodulation will not replace disease-modifying therapy (e.g., RNA-targeted therapy in hereditary transthyretin amyloid polyneuropathy (ATTRv)) but can be positioned earlier as a complementary strategy to preserve function while upstream pathology is treated [66].

A practical research agenda follows from this trajectory. First, trials should prospectively stratify by small-fiber burden and axonal injury markers and prespecify biomarker-anchored response criteria (e.g., ≥X% reduction in pain plus improvement in CNFL or CNFD at 3–6 months), allowing differentiation between pure analgesia and genuine neural recovery [63, 64, 65]. Second, endpoints should broaden beyond pain to include function, sensory-examination domains, and device-relevant metrics (mobility, sleep, activity counts), with adjudicated safety composites that capture infection, lead migration, and wound complications—events that, although infrequent, determine durability and real-world value [41, 43]. Third, implementation science will matter: pathways that define when to pivot from medications to device trials, how to co-manage glycemic and foot-risk care in PDN, and how to maintain post-implant surveillance will likely determine outcomes as much as the waveform itself. Finally, AI-enabled decision support is being explored at both the front end (risk prediction, imaging triage on US/MRN) and the back end (programming suggestions and remote-monitoring dashboards); however, prospective clinical impact, transparency, and governance (bias, privacy, safety monitoring) require careful validation before routine adoption [19, 67, 68].

In parallel with device-based strategies, disease-modifying approaches are emerging. Regenerative medicine is an area to watch. RNA-targeted therapies (siRNA and antisense oligonucleotides) have already reshaped care in hereditary transthyretin amyloidosis (ATTRv) and could serve as a template for disease-modifying strategies in peripheral neuropathies [66]. In Charcot–Marie–Tooth disease (CMT), approaches such as *PMP22* dosage modulation and correction of *GJB1*/connexin-32 pathways are advancing from preclinical to early clinical stages, though practical hurdles persist—efficient delivery to Schwann cells and dorsal root ganglion, durability and re-dosing, and immune reactions [69, 70]. Stem-cell and exosome-based therapies show potential for trophic support, immune modulation, and remyelination but remain investigational with heterogeneous methods and limited evidence; they should stay within clinical trial settings. For translation, standardized outcome batteries (e.g., NIS-LL/mNIS+7, R-ODS, quality of life (QoL) and pain measures, and small-fiber metrics such as corneal confocal microscopy and intraepidermal nerve fiber density) and long-term safety registries are essential [20]. In short, regenerative medicine should be positioned not as incremental analgesia but as a disease-modifying path aimed at target engagement, durable nerve repair, and patient-centered functional recovery.

Taken together, neuromodulation in peripheral neuropathies may shift toward earlier use in well-profiled patients, tighter linkage to biology via quantitative markers, and smarter—potentially closed-loop—platforms intended to maintain benefit in daily life, acknowledging that prospective validation is still evolving. As disease-modifying options expand for selected etiologies, the center of gravity becomes a coordinated continuum: optimize risk factors; escalate to mechanism-matched devices when appropriate; and verify benefit with objective signals of regeneration. In short, relief of pain and restoration of nerve health should be pursued in parallel, not in sequence.

Limitations: As a narrative review, this article does not apply a fully reproducible systematic search strategy or formal risk‑of‑bias grading for every included study, and selection decisions may introduce emphasis bias despite prioritization of guidelines and higher‑level evidence where available. Evidence strength varies across etiologies and modalities; therefore, practice‑facing statements are explicitly qualified when based on limited or heterogeneous data. The diagnostic algorithm is intended to aid triage and communication rather than to replace specialist evaluation or local protocols.

## 8. Conclusions

Peripheral neuropathy encompasses diverse etiologies and pathophysiology, so delayed diagnosis and suboptimal treatment remain common. This narrative review integrates a phenotype‑first, stepwise diagnostic framework—from bedside pattern recognition to core laboratory testing, EDX, and selective escalation to US/MRN, small‑fiber testing, and antibody work‑up when indicated—and it summarizes tiered management options spanning guideline‑supported pharmacotherapy, image‑guided interventions, and selected neuromodulation strategies for carefully selected, refractory cases. Importantly, Fig. 1 is presented as a flexible clinical framework rather than a prescriptive standard; recommendations should be adapted to patient context, local resources, and evolving evidence. Several domains discussed (e.g., some PRF indications, AI‑enabled decision support, and regenerative/exosome‑based therapies) remain investigational and require prospective validation before routine adoption.

## Abbreviations

AN, autoimmune nodopathies; ATTRv, hereditary transthyretin amyloid polyneuropathy; CBA, cell-based assay; CCM, corneal confocal microscopy; CIDP, chronic inflammatory demyelinating polyradiculoneuropathy; CIPN, chemotherapy-induced peripheral neuropathy; CNFL, corneal nerve fiber length; CNFD, corneal nerve fiber density; CNBD, corneal nerve fiber branch density; DRG, dorsal root ganglion; EDX, electrodiagnostics (NCS/EMG); EMG, electromyography; GBS, Guillain–Barré syndrome; IENFD, intraepidermal nerve fiber density; IVIg, intravenous immunoglobulin; mNIS+7/NIS-LL, modified Neuropathy Impairment Score +7/Lower Limbs; MRN, magnetic resonance neurography; NCS, nerve conduction studies; NF155/CNTN1/CASPR1 (pan-NF), nodal/paranodal antigens; NfL, neurofilament light chain; PDN, painful diabetic neuropathy; DPN, diabetic peripheral neuropathy; PE, plasma exchange; PPNI, perioperative peripheral nerve injury; PRF, pulsed radiofrequency; QST, quantitative sensory testing; QSART, quantitative sudomotor axon reflex test; QoL-DN, Quality of Life–Diabetic Neuropathy; SCS, spinal cord stimulation; SNRI/TCA, serotonin–norepinephrine reuptake inhibitors/tricyclic antidepressants; US, ultrasound.

## References

[1] Mirian A, Aljohani Z, Grushka D, Florendo-Cumbermack A (2023). Diagnosis and management of patients with polyneuropathy. *CMAJ: Canadian Medical Association Journal = Journal De L’Association Medicale Canadienne*.

[2] Siao P, Kaku M (2019). A Clinician’s Approach to Peripheral Neuropathy. *Seminars in Neurology*.

[3] Silsby M, Feldman EL, Dortch RD, Roth A, Haroutounian S, Rajabally YA (2023). Advances in diagnosis and management of distal sensory polyneuropathies. *Journal of Neurology, Neurosurgery, and Psychiatry*.

[4] Mauermann ML, Staff NP (2026). Peripheral Neuropathy: A Review. *JAMA*.

[5] Graus F, Vogrig A, Muñiz-Castrillo S, Antoine JCG, Desestret V, Dubey D (2021). Updated Diagnostic Criteria for Paraneoplastic Neurologic Syndromes. *Neurology(R) Neuroimmunology & Neuroinflammation*.

[6] Stino AM, Naddaf E, Dyck PJ, Dyck PJB (2021). Chronic inflammatory demyelinating polyradiculoneuropathy-Diagnostic pitfalls and treatment approach. *Muscle & Nerve*.

[7] Hovaguimian A, Gibbons CH (2011). Diagnosis and treatment of pain in small-fiber neuropathy. *Current Pain and Headache Reports*.

[8] Vinik AI, Ziegler D (2007). Diabetic cardiovascular autonomic neuropathy. *Circulation*.

[9] Watson JC, Dyck PJB (2015). Peripheral Neuropathy: A Practical Approach to Diagnosis and Symptom Management. *Mayo Clinic Proceedings*.

[10] Schmid AB, Fundaun J, Tampin B (2020). Entrapment neuropathies: a contemporary approach to pathophysiology, clinical assessment, and management. *Pain Reports*.

[11] van Doorn PA, Van den Bergh PYK, Hadden RDM, Avau B, Vankrunkelsven P, Attarian S (2023). European Academy of Neurology/Peripheral Nerve Society Guideline on diagnosis and treatment of Guillain-Barré syndrome. *European Journal of Neurology*.

[12] American Diabetes Association Professional Practice Committee (2025). 12. Retinopathy, Neuropathy, and Foot Care: Standards of Care in Diabetes-2025. *Diabetes Care*.

[13] Loprinzi CL, Lacchetti C, Bleeker J, Cavaletti G, Chauhan C, Hertz DL (2020). Prevention and Management of Chemotherapy-Induced Peripheral Neuropathy in Survivors of Adult Cancers: ASCO Guideline Update. *Journal of Clinical Oncology: Official Journal of the American Society of Clinical Oncology*.

[14] Gupta P, Mirman I, Shahar S, Dubey D (2023). Growing Spectrum of Autoimmune Nodopathies. *Current Neurology and Neuroscience Reports*.

[15] Miller LE, Hammert WC, Rekant MS, Fowler JR (2024). Diagnostic Accuracy of Neuromuscular Ultrasound vs. Electrodiagnostic Studies for Carpal Tunnel Syndrome: Systematic Review and Meta-analysis of Paired Accuracy Studies.

[16] Nwawka OK, Adriaensen M, Andreisek G, Drakonaki EE, Lee KS, Lutz AM (2025). Imaging of Peripheral Nerves: AJR Expert Panel Narrative Review. *AJR. American Journal of Roentgenology*.

[17] Nouh MR, Abdel-Naby HM, El Sakka T, El-Shafei M (2025). Peripheral nerve ultrasound: a survival guide for the practicing radiologist with updates. *The Ultrasound Journal*.

[18] Jung JY, Lin Y, Carrino JA (2023). An Updated Review of Magnetic Resonance Neurography for Plexus Imaging. *Korean Journal of Radiology*.

[19] Baal JD, Yoon D, Patel RP, Chin CT, Shah VN (2024). Advanced Imaging of the Peripheral Nerves, From the AJR “How We Do It” Special Series. *AJR. American Journal of Roentgenology*.

[20] Gad H, Elgassim E, Lebbe A, MacDonald RS, Baraka A, Petropoulos IN (2024). Corneal confocal microscopy detects early nerve regeneration after pharmacological and surgical interventions: Systematic review and meta-analysis. *Journal of the Peripheral Nervous System: JPNS*.

[21] Tajabadi Z, Dadkhah PA, Gholami Chahkand MS, Esmaeilpour Moallem F, Karimi MA, Amini-Salehi E (2025). Exploring the role of exosomes in diabetic neuropathy: From molecular mechanisms to therapeutic potential. *Biomedicine & Pharmacotherapy = Biomedecine & Pharmacotherapie*.

[22] Lukashenko MV, Gavrilova NY, Bregovskaya AV, Soprun LA, Churilov LP, Petropoulos IN (2021). Corneal Confocal Microscopy in the Diagnosis of Small Fiber Neuropathy: Faster, Easier, and More Efficient Than Skin Biopsy?. *Pathophysiology: the Official Journal of the International Society for Pathophysiology*.

[23] Ryan CS, Conlee EM, Sharma R, Sorenson EJ, Boon AJ, Laughlin RS (2019). Nerve conduction normal values for electrodiagnosis in pediatric patients. *Muscle & Nerve*.

[24] Dyck PJ, Kincaid JC, Dyck PJB, Chaudhry V, Goyal NA, Alves C (2017). Assessing mNIS+7I⁢o⁢n⁢i⁢s and international neurologists’ proficiency in a familial amyloidotic polyneuropathy trial. *Muscle & Nerve*.

[25] Smith EML, Cohen JA, Pett MA, Beck SL (2010). The reliability and validity of a modified total neuropathy score-reduced and neuropathic pain severity items when used to measure chemotherapy-induced peripheral neuropathy in patients receiving taxanes and platinums. *Cancer Nursing*.

[26] Lauria G, Hsieh ST, Johansson O, Kennedy WR, Leger JM, Mellgren SI (2010). European Federation of Neurological Societies/Peripheral Nerve Society Guideline on the use of skin biopsy in the diagnosis of small fiber neuropathy. Report of a joint task force of the European Federation of Neurological Societies and the Peripheral Nerve Society. *European Journal of Neurology*.

[27] Price R, Smith D, Franklin G, Gronseth G, Pignone M, David WS (2022). Oral and Topical Treatment of Painful Diabetic Polyneuropathy: Practice Guideline Update Summary: Report of the AAN Guideline Subcommittee. *Neurology*.

[28] Finnerup NB, Attal N, Haroutounian S, McNicol E, Baron R, Dworkin RH (2015). Pharmacotherapy for neuropathic pain in adults: a systematic review and meta-analysis. *The Lancet. Neurology*.

[29] Balanaser M, Carley M, Baron R, Finnerup NB, Moore RA, Rowbotham MC (2023). Combination pharmacotherapy for the treatment of neuropathic pain in adults: systematic review and meta-analysis. *Pain*.

[30] Derry S, Rice AS, Cole P, Tan T, Moore RA (2017). Topical capsaicin (high concentration) for chronic neuropathic pain in adults. *The Cochrane Database of Systematic Reviews*.

[31] Narouze S, Benzon HT, Provenzano D, Buvanendran A, De Andres J, Deer T (2018). Interventional Spine and Pain Procedures in Patients on Antiplatelet and Anticoagulant Medications (Second Edition): Guidelines From the American Society of Regional Anesthesia and Pain Medicine, the European Society of Regional Anaesthesia and Pain Therapy, the American Academy of Pain Medicine, the International Neuromodulation Society, the North American Neuromodulation Society, and the World Institute of Pain. *Regional Anesthesia and Pain Medicine*.

[32] Mahadevan D, Attwal M, Bhatt R, Bhatia M (2016). Corticosteroid injection for Morton’s neuroma with or without ultrasound guidance: a randomised controlled trial. *The Bone & Joint Journal*.

[33] Lizano-Díez X, Ginés-Cespedosa A, Alentorn-Geli E, Pérez-Prieto D, González-Lucena G, Gamba C (2017). Corticosteroid Injection for the Treatment of Morton’s Neuroma: A Prospective, Double-Blinded, Randomized, Placebo-Controlled Trial. *Foot & Ankle International*.

[34] Ashkenazi A, Matro R, Shaw JW, Abbas MA, Silberstein SD (2008). Greater occipital nerve block using local anaesthetics alone or with triamcinolone for transformed migraine: a randomised comparative study. *Journal of Neurology, Neurosurgery, and Psychiatry*.

[35] Dilli E, Halker R, Vargas B, Hentz J, Radam T, Rogers R (2015). Occipital nerve block for the short-term preventive treatment of migraine: A randomized, double-blinded, placebo-controlled study. *Cephalalgia: an International Journal of Headache*.

[36] Ambrosini A, Vandenheede M, Rossi P, Aloj F, Sauli E, Pierelli F (2005). Suboccipital injection with a mixture of rapid- and long-acting steroids in cluster headache: a double-blind placebo-controlled study. *Pain*.

[37] Benzon HT, Elmofty D, Shankar H, Rana M, Chadwick AL, Shah S (2025). Use of corticosteroids for adult chronic pain interventions: sympathetic and peripheral nerve blocks, trigger point injections - guidelines from the American Society of Regional Anesthesia and Pain Medicine, the American Academy of Pain Medicine, the American Society of Interventional Pain Physicians, and the International Pain and Spine Intervention Society. *Regional Anesthesia and Pain Medicine*.

[38] Lam KHS, Hung CY, Chiang YP, Onishi K, Su DCJ, Clark TB (2020). Ultrasound-Guided Nerve Hydrodissection for Pain Management: Rationale, Methods, Current Literature, and Theoretical Mechanisms. *Journal of Pain Research*.

[39] Petersen EA, Stauss TG, Scowcroft JA, Jaasma MJ, Brooks ES, Edgar DR (2023). Long-term efficacy of high-frequency (10 kHz) spinal cord stimulation for the treatment of painful diabetic neuropathy: 24-Month results of a randomized controlled trial. *Diabetes Research and Clinical Practice*.

[40] Blackburn AZ, Chang HH, DiSilvestro K, Veeramani A, McDonald C, Zhang AS (2021). Spinal Cord Stimulation via Percutaneous and Open Implantation: Systematic Review and Meta-Analysis Examining Complication Rates. *World Neurosurgery*.

[41] Petersen EA, Stauss TG, Scowcroft JA, Brooks ES, White JL, Sills SM (2021). Effect of High-frequency (10-kHz) Spinal Cord Stimulation in Patients With Painful Diabetic Neuropathy: A Randomized Clinical Trial. *JAMA Neurology*.

[42] Kapural L, Yu C, Doust MW, Gliner BE, Vallejo R, Sitzman BT (2015). Novel 10-kHz High-frequency Therapy (HF10 Therapy) Is Superior to Traditional Low-frequency Spinal Cord Stimulation for the Treatment of Chronic Back and Leg Pain: The SENZA-RCT Randomized Controlled Trial. *Anesthesiology*.

[43] Hoelzer BC, Bendel MA, Deer TR, Eldrige JS, Walega DR, Wang Z (2017). Spinal Cord Stimulator Implant Infection Rates and Risk Factors: A Multicenter Retrospective Study. *Neuromodulation: Journal of the International Neuromodulation Society*.

[44] Mattie R, Schneider BJ, Miller DC, Popescu A, Smith CC, McCormick ZL (2022). Factfinders for patient safety: Antibiotics for disc access and spinal cord stimulation trials. *Interventional Pain Medicine*.

[45] Park D, Chang MC (2022). The mechanism of action of pulsed radiofrequency in reducing pain: a narrative review. *Journal of Yeungnam Medical Science*.

[46] Jorge DDMF, Huber SC, Rodrigues BL, Da Fonseca LF, Azzini GOM, Parada CA (2022). The Mechanism of Action between Pulsed Radiofrequency and Orthobiologics: Is There a Synergistic Effect?. *International Journal of Molecular Sciences*.

[47] Yuba T, Koyama Y, Uematsu H, Takahashi A, Matsuda Y, Fujino Y (2025). Elucidation of the treatment mechanism of pulsed radiofrequency based on its antiinflammatory effects. *Scientific Reports*.

[48] Chen LC, Ho CW, Sun CH, Lee JT, Li TY, Shih FM (2015). Ultrasound-Guided Pulsed Radiofrequency for Carpal Tunnel Syndrome: A Single-Blinded Randomized Controlled Study. *PloS One*.

[49] Cohen SP, Peterlin BL, Fulton L, Neely ET, Kurihara C, Gupta A (2015). Randomized, double-blind, comparative-effectiveness study comparing pulsed radiofrequency to steroid injections for occipital neuralgia or migraine with occipital nerve tenderness. *Pain*.

[50] Deniz S, Purtuloglu T, Tekindur S, Cansız KH, Yetim M, Kılıckaya O (2015). Ultrasound-guided pulsed radio frequency treatment in Morton’s neuroma. *Journal of the American Podiatric Medical Association*.

[51] Batistaki C, Madi AI, Karakosta A, Kostopanagiotou G, Arvaniti C (2021). Pulsed Radiofrequency of the Occipital Nerves: Results of a Standardized Protocol on Chronic Headache Management. *Anesthesiology and Pain Medicine*.

[52] Mücke M, Phillips T, Radbruch L, Petzke F, Häuser W (2018). Cannabis-based medicines for chronic neuropathic pain in adults. *The Cochrane Database of Systematic Reviews*.

[53] Cohen SP, Bhatia A, Buvanendran A, Schwenk ES, Wasan AD, Hurley RW (2018). Consensus Guidelines on the Use of Intravenous Ketamine Infusions for Chronic Pain From the American Society of Regional Anesthesia and Pain Medicine, the American Academy of Pain Medicine, and the American Society of Anesthesiologists. *Regional Anesthesia and Pain Medicine*.

[54] Barohn RJ, Gajewski B, Pasnoor M, Brown A, Herbelin LL, Kimminau KS (2021). Patient Assisted Intervention for Neuropathy: Comparison of Treatment in Real Life Situations (PAIN-CONTRoLS): Bayesian Adaptive Comparative Effectiveness Randomized Trial. *JAMA Neurology*.

[55] Chui J, Murkin JM, Posner KL, Domino KB (2018). Perioperative Peripheral Nerve Injury After General Anesthesia: A Qualitative Systematic Review. *Anesthesia and Analgesia*.

[56] Lam KK, Soneji N, Katzberg H, Xu L, Chin KJ, Prasad A (2020). Incidence and etiology of postoperative neurological symptoms after peripheral nerve block: a retrospective cohort study. *Regional Anesthesia and Pain Medicine*.

[57] Brull R, Hadzic A, Reina MA, Barrington MJ (2015). Pathophysiology and Etiology of Nerve Injury Following Peripheral Nerve Blockade. *Regional Anesthesia and Pain Medicine*.

[58] O’Flaherty D, McCartney CJL, Ng SC (2018). Nerve injury after peripheral nerve blockade-current understanding and guidelines. *BJA Education*.

[59] Abdallah FW, Chan VWS (2014). Monitoring intraneural needle injection: work in progress. *Anesthesia and Analgesia*.

[60] Costa F, Pascarella G, Del Buono R, Strumia A, Schiavoni L, Mattei A (2022). Opening injection pressure as a part of multimodal monitoring to detect intraneural injections. *Regional Anesthesia and Pain Medicine*.

[61] Chang J, Bhandari L, Messana J, Alkabbaa S, Hamidian Jahromi A, Konofaos P (2022). Management of Tourniquet-Related Nerve Injury (TRNI): A Systematic Review. *Cureus*.

[62] Lirk P, Brummett CM (2019). Regional anaesthesia, diabetic neuropathy, and dexmedetomidine: a neurotoxic combination?. *British Journal of Anaesthesia*.

[63] Gad H, Petropoulos IN, Khan A, Ponirakis G, MacDonald R, Alam U (2022). Corneal confocal microscopy for the diagnosis of diabetic peripheral neuropathy: A systematic review and meta-analysis. *Journal of Diabetes Investigation*.

[64] Maalmi H, Strom A, Petrera A, Hauck SM, Strassburger K, Kuss O (2023). Serum neurofilament light chain: a novel biomarker for early diabetic sensorimotor polyneuropathy. *Diabetologia*.

[65] Määttä LL, Andersen ST, Parkner T, Hviid CVB, Bjerg L, Kural MA (2024). Longitudinal Change in Serum Neurofilament Light Chain in Type 2 Diabetes and Early Diabetic Polyneuropathy: ADDITION-Denmark. *Diabetes Care*.

[66] Coelho T, Marques W, Dasgupta NR, Chao CC, Parman Y, França MC (2023). Eplontersen for Hereditary Transthyretin Amyloidosis With Polyneuropathy. *JAMA*.

[67] Beste NC, Jende J, Kronlage M, Kurz F, Heiland S, Bendszus M (2024). Automated peripheral nerve segmentation for MR-neurography. *European Radiology Experimental*.

[68] Baskozos G, Themistocleous AC, Hebert HL, Pascal MMV, John J, Callaghan BC (2022). Classification of painful or painless diabetic peripheral neuropathy and identification of the most powerful predictors using machine learning models in large cross-sectional cohorts. *BMC Medical Informatics and Decision Making*.

[69] Caballé RB, Bortolozzi M (2024). New perspectives for gene therapy of the X-linked form of Charcot-Marie-Tooth disease. *Molecular Therapy. Methods & Clinical Development*.

[70] Zhao HT, Damle S, Ikeda-Lee K, Kuntz S, Li J, Mohan A (2018). PMP22 antisense oligonucleotides reverse Charcot-Marie-Tooth disease type 1A features in rodent models. *The Journal of Clinical Investigation*.

